# Enhancing resilience and mental well-being among paediatric nurses: a systematic review of effective strategies and implementation challenges

**DOI:** 10.1007/s00431-025-06647-y

**Published:** 2025-11-24

**Authors:** Godfrey Mbaabu Limungi, Klara Simon, Mohammed Elmadani, Mesmar Amer, Osama Hamad, Livia Tóth, Eva Horvath, Ummay Soumayia Islam, Nancy Ntinyari, Orsolya Mate

**Affiliations:** 1 Doctoral School of Health Sciences, Faculty of Health Sciences, University of Pécs, Pécs, Hungary; 2 Faculty of Health Sciences, University of Pécs, Pecs, Hungary

**Keywords:** Paediatric nurses, Resilience, Mental well-being, Intervention strategies, Implementation challenges

## Abstract

Paediatric nurses frequently experience emotional and psychological stress due to the complexities of family-centred care. These pressures can compromise their mental health and resilience, which are critical for sustaining their well-being and delivering high-quality paediatric care. This systematic review examined strategies designed to enhance resilience and mental well-being among paediatric nurses and explored barriers to their implementation. The review protocol was registered with PROSPERO (CRD42024537933) and conducted according to PRISMA guidelines. Comprehensive searches were performed across Scopus, Web of Science, Medline, Embase, CINAHL, PubMed, and Google Scholar. Eligible studies included quantitative, qualitative, and mixed-methods research focusing on resilience-building strategies among paediatric nurses. The Mixed Methods Appraisal Tool (MMAT) was used for quality assessment, and data were synthesized through qualitative content analysis. From 990 records, six studies (total *N* = 486) met the inclusion criteria. Interventions were evaluated using validated tools such as the Professional Quality of Life Scale (ProQOL), Connor-Davidson Resilience Scale (CD-RISC), Perceived Stress Scale (PSS), and Self Compassion Scale (SCS). Four key strategies were identified: mindfulness practices, education workshops, mobile health (mHealth) applications, and Employee Assistance Programs (EAPs). Mindfulness interventions improved emotional regulation and resilience; education workshops reduced stress and promoted coping; mHealth apps increased perceived resilience, and EAPs provided counselling support with measurable gains. *Conclusion*: Resilience-enhancing interventions such as mindfulness practices, education workshops, mHealth applications, and EAPs appear promising for improving the well-being of paediatric nurses. However, the current evidence base is limited and diverse, meaning these strategies should be considered provisional and applied with caution. Future multicentre longitudinal research is needed to establish their sustained effectiveness and develop interventions that are sustainable and easily integrated into paediatric healthcare practice.

**Table:** 

**What is Known:** • *Strategies to enhance resilience and mental well-being among nurses have been studied, but most evidence is fragmented across general nursing populations.* • *Few studies have examined paediatric nurses specifically, despite the unique emotional and clinical demands of paediatric care.*
**What is New:** • *This review provides the first focused synthesis of evidence on resilience-enhancing strategies among paediatric nurses.* • *Mindfulness-based interventions demonstrated the strongest and most consistent evidence for improving resilience, reducing stress, and enhancing emotional regulation.*

**Supplementary Information:**

The online version contains supplementary material available at 10.1007/s00431-025-06647-y.

## Introduction

Paediatric nursing presents unique challenges that demand high levels of physical, emotional, and mental resilience [1]. Nurses in paediatric unit care for critically sick children and their families, often under high-pressure conditions, which places them at risk of negative mental outcomes. The intensity of these roles exposes nurses to high levels of occupational stress and emotional burden as they deal with complex patient needs, distressed families, and the pressures of the healthcare system [2]. Maintaining the mental well-being of paediatric nurses is essential not only for the nurses themselves but also for the quality of patient care and family support [3–5].

For the purpose of this review, paediatric nurses are defined as registered nurses providing direct care to children in inpatient or outpatient paediatric settings, regardless of whether they hold advanced paediatric qualifications. Resilience refers to the dynamic capacity of an individual to adapt positively and recover from adversity, stress or trauma while maintaining personal and professional functioning [6, 7]. Resilience is not merely the absence of psychological symptoms, but it involves active coping, emotional regulation, and the ability to sustain well-being despite workplace pressures.

Resilience plays a crucial role in protecting nurses from adverse mental health outcomes [6, 7]. Existing literature indicates that paediatric nurses often experience moderate to high occupational stress and lower resilience scores compared to general nursing population, which can adversely affect their well-being, professional performance, and patient safety [8–10]. Reported mean Perceived Stress Scale (PSS) scores for paediatric nurses range from 20 to 26, indicating moderate stress levels. For instance, in an Iranian clinical trial using the Kobasa Hardness Inventory and Perceived Stress Scale (PSS), paediatric nurses reported a baseline PSS mean of 26.54 (SD 7.59) [22]. Similarly, among neonatal intensive care unit (NICU) staff, pre-interventional Professional Quality of Life (ProQOL) subscale means were as follows: compassion satisfaction = 39.4 (SD 5.6), burnout = 24.4 (SD 6.0), and secondary traumatic stress 24.5 (SD 6.5) [19]. In a study of oncology and palliative paediatric staff, rescaled Work Stressors Scale means reached 60.8 for child-related stressors, 52.6 for organizational stressors, and 47.1 for parent-related factors [8]. Reduced resilience is associated with psychological symptoms, increased risk of medical errors, lower quality of care, and increased patient dissatisfaction [11, 12]. In addition, stress and emotional exhaustion contribute to decreased job satisfaction, nurse turnover, and understaffing, further challenging paediatric healthcare delivery [13–15]. Experienced nurses may leave bedside care or transition to other specialties, impacting institutional capacity and continuity of care [15].

Despite research on nurse resilience and mental well-being in other areas, such as psychiatric or critical care nursing, comprehensive studies focusing specifically on paediatric nurses remain limited. Existing literature often addresses individual components of stress or resilience but lacks integration regarding effective strategies designed for the paediatric context. Understanding these interventions is critical given the unique emotional and professional demands placed on paediatric nurses.

Therefore, this systematic review aimed to explore effective strategies for enhancing resilience and mental well-being among paediatric nurses and to examine the challenges associated with implementing these strategies. Identifying and understanding these strategies is vital for developing supportive plans that strengthen resilience, promote mental wellness among paediatric nurses, and ensure high-quality paediatric care.

## Methodology

### Research question

What are the effective strategies implemented in paediatric healthcare units to promote resilience and mental well-being among paediatric nurses, and what are the perceived challenges to their implementation?

### Protocol and registration

The protocol was registered in the international register of the Prospective Register for Systematic Reviews (PROSPERO) on 1 st May 2024, with registration no. CRD42024537933. The review adhered to the guidelines set by the Preferred Reporting Items for Systematic Reviews and Meta-Analyses (PRISMA) statement [16]. Prior to final submission, the search strategy was rerun and refreshed on 5th of May 2025 to ensure inclusion of the most recent and relevant literature.

### Eligibility criteria

The review included all studies incorporating observational designs (quantitative, qualitative, or mixed methods) and interventional designs (Randomized Clinical Trials or Non-Randomized experimental studies) published in English in peer-reviewed journals and focusing on strategies for promoting resilience and mental well-being among pediatric nurses. Studies that focused on paediatric nurses of all genders and any specialization as the study population or subpopulation were included. Discussion papers, dissertations, theses, commentaries, editorials, systematic reviews, scoping reviews, meta-analyses, and literature reviews were excluded from this review. Systematic reviews and other secondary research were excluded because this review sought to synthesize primary evidence. Including them could have introduced duplication of the data already captured in the original studies. Theses and dissertations were excluded not only because they often undergo limited peer review but also due to challenges in retrieval, variable methodological quality, and incomplete reporting. Restricting the review to peer-reviewed primary research ensured inclusion of studies with greater methodological rigor.

### Databases and search strategies

Two research team members independently searched the following databases according to eligibility criteria: Scopus, Medline, Web of Science, CINAHL, Embase, PubMed, and Google Scholar. The initial search was conducted between 1 st and 7th May 2024 and updated on 5th May 2025 to capture recent publications. The search was without any restrictions on publication date to ensure comprehensive inclusion. The search strategy included (“paediatric nurse” OR “pediatric nurse” OR “paediatric nurses” OR “pediatric nurses” OR “children’s nurses” OR “paediatric nursing staff” OR “pediatric nursing staff”) AND (“resilience” OR “adaptability” OR “perseverance”) AND (“mental well-being” OR “mental wellness” OR “mental health”) AND (“strategies” OR “interventions” OR “programs” OR “support programs” OR “coping mechanisms”). Articles from these databases included studies in which paediatric nurses were either the primary sample or a part of the broader multidisciplinary sample. In this review, paediatric nurses were defined as any nurse working in the paediatric unit, regardless of whether they had specialized training in paediatric nursing. The full electronic search for each database is provided in Supplementary Material 1.

### Selection of sources of evidence

**Fig. 1:**
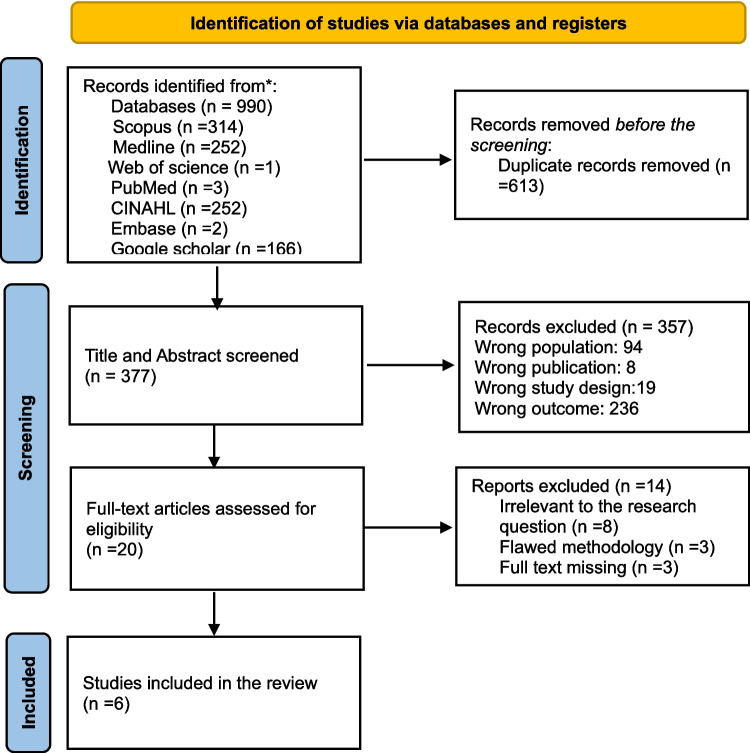
PRISMA flow diagram of study selection

After retrieving the articles from the databases, they were all uploaded to the Rayyan software, and duplicates were removed before the review was conducted by the research team. Two reviewers from the research team evaluated independently all the papers by titles and abstracts to determine if they met the selection criteria through a blind process. The selected citations were subsequently reviewed as full-text articles for inclusion, again involving a blind process. Arising conflicts were resolved by discussion. The selection process is depicted in Fig. 1, while reasons for exclusion at the full-text screening stage are provided in Supplementary Material 2.

### Quality appraisal

The quality of the included studies was assessed using the Mixed Methods Appraisal Tool (MMAT) [17]. Each study was evaluated against appropriate methodological criteria and categorized as having a low risk of bias (met all or most of the MMAT quality criteria), some concern of bias (met several but not all MMAT quality criteria), or a high risk of bias (met few MMAT quality criteria). In addition to assessing risk of bias, the overall quality and strength of evidence were also assessed. Quality of evidence was rated as high, moderate, or low based on the methodological rigor of the study and the consistency of its findings across multiple sources. Strength of recommendation was categorized as strong, moderate, or weak. This categorization did not follow the formal GRADE framework but was based on the methodological rigor of the included studies, the number and consistency of studies reporting similar findings, and the practical relevance and feasibility of implementing the strategy in paediatric nursing settings. “Strong” referred to strategies supported by multiple methodologically sound studies, with consistent findings and clear practical applicability. “Moderate” referred to strategies supported by a limited number of studies or with some concerns regarding consistency or feasibility. “Weak” referred to studies with minimal evidence. Two research team members independently conducted the quality assessments, and in cases of disagreement, a third reviewer was consulted to reach consensus. A detailed MMAT scoring table per study is provided in Supplementary Material 3, while the grading of the evidence is illustrated in Table 2.

### Data charting process

This systematic review used a data extraction table, developed by the principal researcher to extract data from the included studies. Before data extraction, this tool was pretested, and the research team agreed that it could capture the intended data. The data that were extracted included study details (authors, publication year, title, study design, and country where the study was conducted), description of paediatric units involved, types of strategies implemented, duration and frequency of implementation, outcomes of these strategies, any challenges or barriers encountered during implementation, views/opinion from nurses regarding the effectiveness of the strategies, conclusions, recommendations, and funding sources if reported. Where information relevant to the research question was missing or unclear, the available data were extracted as presented in the publication, and gaps were documented in the extraction table as “not reported.” Study authors were not contacted for additional information due to time constraints.

### Data synthesis and analysis

Given the heterogeneity in study designs, a narrative synthesis approach was adopted. Data were sub grouped as pre-specified by study design, outcome domain and intervention type, before being synthesized qualitatively through content analysis to identify key strategies and implementation challenges [18]. Findings from quantitative studies were integrated with those from qualitative studies by identifying common themes and mapping them across the study design. For example, intervention outcomes such as reduced stress or improvement in resilience were compared with qualitative reports of nurses’ perceptions of effectiveness.

## Results

### Description of the search process and characteristics of the included studies

The initial database search yielded 990 potential articles, from which 377 publications were selected for consideration after 614 duplicates were removed. In all the databases, these articles had been published between 1991 and 2024, with most of the publications occurring between 2018 and 2024. Title and abstract screening was performed, and 357 publications were excluded due to various reasons, as illustrated in Fig. 1. The selected 20 articles proceeded for full-text screening. Applying the inclusion and exclusion criteria, 14 studies were excluded. Therefore, six studies were deemed relevant and included for data extraction and analysis. The characteristics of these studies are detailed in Table 1. Clinical trial studies were the most prevalent (*n* = 5), with four studies using non-randomized study design, while one study used randomized study design. Additionally, one study employed a cross-sectional study design with a descriptive qualitative approach. Only two studies used paediatric nurses as a subsample from the large sample of paediatric staff. Paediatric nurses from the paediatric outpatient unit were involved in one study while most studies relied on nurses from the inpatient units. Most of the included studies (66.7%, *n* = 4) were conducted in the USA, while the rest were conducted in Australia (16.7%, *n* = 1) and Iran (16.7%, *n* = 1). Funding sources were disclosed in one study (Peterson et al., 2024, supported by the Myrtie Fulton Endowment Mentorship Award). The remaining five studies did not report funding information.

**Table 1:** Characteristics of the included studies

Author (s), year	Country	Study design	Paediatric unit	Intervention type	Key outcome	Funding source
Slater et al., 2018	Australia	Clinical trial (non-randomized	Paediatric oncology, haematology, palliative care	Mindfulness practices. Education workshop, EAPs	Improved morale, communication, and resilience	Not reported
Zadeh et al., 2012	USA	Clinical trial (non-randomized)	Outpatient and inpatient departments	Education workshop	Increased empowerment, emotional support and engagement	Not reported
Peterson et al., 2024	USA	3-arm pre-post interventional	Neonatal intensive unit	mHealth, gratitude and mindfulness	Reduced burnout, reduced secondary stress, increased happiness	Myrtie Fulton Endowment Mentorship Award
Franco and Christie, 2021	USA	Quasi-experimental design	General paediatric	One day self-compassion training	Increased resilience reduced burnout and stress	Not reported
Wei et al., 2020	USA	Cross-sectional (qualitative descriptive study)	Paediatric intensive care unit and intermediate care unit	Self-care; meaning making, interpersonal connections, emotional hygiene, positivity	Meaningful work and connection were important	Not reported
Sadeghpour et al., 2021	Iran	Clinical trial (RCT)	Paediatric inpatient units	Education program and exercise using Kobasa and Maddi hardiness model	Increased hardness, reduced stress	Not reported

### Grading of evidence

Only six studies that passed the quality appraisal were included for data extraction and synthesis. These studies were graded based on the quality of evidence and strength of recommendation. Strategies supported by four or more studies with acceptable methodological quality were graded as high quality and interpreted as providing strong support. Strategies supported by two or three studies were graded as moderate and interpreted as providing conditional support, while those supported by a single study were graded as low and interpreted as providing weak support. A summary of this grading is presented in Table 2.

**Table 2:** Grading of evidence

Strategy	Number of studies	Quality of evidence	Strength of recommendation	Explanation
Mindfulness practices	4	High	Strong	Consistent improvements in resilience and stress reduction across Slater et al. (2018), Franco and Christie (2021), Peterson et al. (2024), Wei et al. (2020)
Education workshops	4	High	Strong	Demonstrated significant benefits in resilience, empowerment and morale (Zadeh et al. 2012; Franco & Christie 2021; Sadeghpour et al. 2021; Slater et al. 2018)
mHealth applications	1	Low	Weak	Evaluated only by Peterson et al. (2024); showed promising quantitative results but limited replication
Employee Assistance Programs	1	Low	Weak	Explored in Slater et al. (2018); perceived helpful, but limited by uptake and integration challenges

### Effective strategies for enhancing resilience and mental well-being in paediatric nurses

The six studies included in this review revealed a range of strategies for enhancing resilience and improving the mental well-being of paediatric nurses. These strategies were grouped into four categories, including mindfulness practices, education workshops, mobile health (mHealth) applications, and Employee Assistance Programs (EAPs). Each of these strategies varied in their approach, delivery, and measured outcome. Mindfulness practices emerged as a widely adopted strategy featured in four of the six included studies [1, 8, 19, 20].

These interventions were critical in helping nurses to manage stress, increase self-awareness, and cultivate emotional regulation. Common components included meditation and breathing exercises, often delivered in brief structured Sects. (15 min of mindfulness practices over at least 6 weeks). These studies reported improvements in self-compassion, happiness, resilience, and reduction in burnout, stress, and secondary traumatic stress. These outcomes were measured using both qualitative interviews and validated quantitative tools such as Professional Quality of Life. The consistency of these findings supports mindfulness as a highly effective and evidence-based approach, though the ProQOL scale, Perceived Affective Well-Being scale (PAVS), and the Subjective Happiness Scale (SHS). The overall effectiveness of mindfulness practices was rated high, though sustained engagement was noted as a practical concern due to workload and time constraints.

Four studies [8, 20–22] employed education workshops focusing on psychological resilience, compassion, and stress management. The sessions by Sadeghpour et al. [2021] incorporated theoretical models such as the Kobasa and Maddi hardness model and included interactive. These sessions included interactive components like role playing, reflective practices, and peer discussion. Outcomes included increased morale, compassion satisfaction, empowerment, and resilience. Effectiveness was measured by using both self-report surveys and standardized scales. Education workshops were generally well received and demonstrated high effectiveness. However, scheduling difficulties, particularly related to shift work, limited participation.

Peterson et al. [2024] was the only study to utilize a mobile health (mHealth) application, where a three-arm interventional study showed that a mobile-based gratitude and mindfulness tool led to statistically significant reductions in burnout and secondary traumatic stress, alongside increased happiness and resilience. The high effectiveness of mHealth interventions lies in their accessibility and real-time support features, which include guided meditations, resilience education modules, and peer support forums. Despite its promise, the study acknowledged that user engagement may be hindered by technical challenges and variability in digital literacy among participants.

Only Slater et al. [2018] evaluated the impact of EAPs. The study found that EAPs improved communication and perceived psychological support among paediatric nurses. Though qualitative in nature, these findings indicate moderate effectiveness, highlighting the potential of EAPs as supplementary resilience-promoting resources. The effectiveness of these strategies is illustrated in Table 3, which compares the effectiveness of various strategies.

**Table 3:** Effectiveness of various strategies

Strategy	Studies supporting	Evidence of effectiveness	Outcome measures used	Overall effectiveness	Limitation
Mindfulness Practices	Slater et al. (2018) Franco and Christie (2021) Peterson et al. (2024) Wei et al. (2020)	Improved morale (qualitative) ↑ Resilience, ↓ burnout (*p* < 0.001) ↓ Burnout (*p* = 0.03) ↑ Happiness (*p* < 0.001)	Pre-post surveys Self-compassion scale Validated scales: ProQOL, PAVS, SHS Interviews	High. Consistently effective in both qualitative and quantitative studies	Requires consistent practice; scheduling issues
Education workshops	Zadeh et al. (2012), Franco and Christie (2021), Sadeghpour et al. (2021), Slater et al. (2018)	↑Engagement and support ↑Resilience and compassion ↓ Stress (*p* < 0.05), ↑hardiness (*p* < 0.02) ↑Engagement, satisfaction and morale	Pre-post surveys self-compassion scale Kobasa and Maddi hardiness scale Pre-post surveys	High. Broadly effective	Attendance challenges due to shift work; sustainability
mHealth applications	Peterson et al. (2024)	↓ Burnout (*t* = 2.30, *p* = 0.03), ↑ happiness (*t* = − 3.72, *p* < 0.001), ↓Secondary traumatic stress(*p* = 0.04) ↑ Resilience	Validated scales: ProQOL, SHS, PAVS; pre-post intervention scores	High — strong quantitative support	Technical barriers
Employee Assistance Programs (EAPs)	Slater et al. (2018)	Improved communication and perceived support	Qualitative feedback; staff satisfaction surveys	Moderate — perceived as helpful, but needs more empirical support	Lack of integration into organizational culture

## Challenges in implementing resilience-enhancing strategies among paediatric nurses

Despite their promise, implementing resilience-enhancing strategies in paediatric nursing practice is faced with several challenges. These barriers, as reported across the reviewed studies, affect the accessibility, sustainability, and integration of the interventions. One of the main challenges involves the sustainability of mindfulness practices. According to Slater et al. [2018], although highly effective, mindfulness practices require consistency and long-term practice, which is often difficult to maintain in a high-pressure clinical environment, especially in paediatric nursing [8]. Staff shortages and time constraints further limit nurses’ ability to engage regularly in mindfulness sessions, especially in paediatric units that lack mindfulness programs in their routine schedules [1, 8].

Educational workshops, though broadly effective, face significant scheduling and participation barriers. Zadeh et al. [2012] and Sadeghpour et al. [2021] noted that rotating shifts night duties and long working hours make it difficult for paediatric nurses to attend structured in-person sessions consistently. These workload pressures can lead to burnout which in turn reduces motivation to engage in resilience training. Moreover, resource limitations were identified as barriers to sustaining educational initiatives in paediatric units [21, 22].

Technological barriers present another layer of difficulty in the implementation of mHealth interventions. As reported by Peterson et al. [2024], while app-based mindfulness and gratitude programs offer flexibility and accessibility, their success depends heavily on stable internet access and user comfort with digital tools. Some paediatric nurses reported reluctance to engage due to limited digital literacy or inadequate institutional support [19].

Employee Assistance Programs (EAPs), though perceived positively by some participants, also face organizational and cultural challenges. Slater et al. [2018] highlighted low program uptake and limited integration into workplace culture as significant issues. Concerns about confidentiality and perceive stigma associated with seeking psychological help were cited as barriers that reduced the utilization and overall impact of EAPs [8].

## Discussion

This systematic review synthesized evidence from six studies examining strategies to enhance resilience among paediatric nurses. Four approaches emerged: mindfulness practices, education workshops, mobile health (mHealth) applications, and Employee Assistance Programs (EAPs). While each demonstrated potential benefits for improving resilience among paediatric nurses, the evidence base remains small and heterogeneous, with only six studies employing varied designs.

Mindfulness practices emerged as the most evaluated intervention, with four of the six studies [1, 8, 19, 20] reporting improvement in emotional regulation, stress management, and overall mental wellness among paediatric nurses. These findings suggest that mindfulness programs are both feasible and effective in promoting mental wellness, although sustainability and consistent participation remain practical challenges due to workload and time constraints. These conclusions are consistent with broader evidence in healthcare populations such as the meta-analysis by Wang et al. [2023] which confirmed the general efficacy of mindfulness-based interventions in reducing stress and burnout [23].

Education workshops were also implemented with four studies [8, 20–22] and consistently demonstrated improvement in empowerment, compassion satisfaction, and resilience. In particular, programs based on Kobasa and Maddi Hardness Models appeared effective in helping paediatric nurses develop adaptive coping strategies and self-care techniques [22]. Despite these benefits, several studies reported barriers to sustained participation such as shift work, work load, and limited organizational support [21, 22]. These findings suggest that workshop-based interventions are valuable but require adaptation for paediatric contexts to ensure accessibility and continuity. The findings concur with a meta-analysis by Han and Yeun [2023], which found that psychological interventions significantly improved resilience among nurses, both immediately post-intervention and within 3 months [4].

The integration of technology, particularly mHealth applications, though investigated in a single study, demonstrated a significant reduction in burnout and secondary traumatic stress and improvement in happiness and resilience. These findings suggest that mobile platforms may represent a flexible means of delivering resilience interventions, particularly for nurses working variable shifts. However, challenges such as variable digital literacy among paediatric nurses and technical issues limit the widespread adoption and sustained use of these tools [19], which must be addressed before broader adoption in paediatric setting.

Employee Assistance Programs (EAPs), assessed in one study [8], showed perceived improvement in communication and psychological support. However, uptake was limited often due to concern about confidentiality, stigma, and lack of integration within the organizational culture [8]. These findings indicate that while EAPs hold potential as supplementary support mechanism, their success depends on institutional endorsement and normalization within paediatric care environments.

## Implications for practice

The findings of this review suggest potential resilience-enhancing strategies that could be applied in paediatric settings. Mindfulness practices, even in short, regular sessions, may be incorporated into daily routine schedules provided that organizational support is available. Evidence from broader nursing population, such as the randomized clinical trial by Alhawatmeh et al. [2022] [24], reinforces that guided imagery and progressive muscle relaxation techniques related to mindfulness significantly reduce stress and emotional symptoms among nurses. Educational workshops, particularly those based on established theoretical models, may help build resilience but require adaptation to accommodate shift work and workload demands. Mobile health applications (mHealth) offer a flexible tool that may be especially suitable for staff working irregular schedules, though technical and training barriers must be addressed. EAPs remain underutilized but could be strengthened through awareness campaigns and assurances of confidentiality. Across all strategies, leadership support, allocation of protected time, and integration in the organizational culture are critical for sustainability.

Future research should aim to strengthen the evidence base by utilizing longitudinal designs to capture the sustained impact of resilience-building strategies. Additionally, qualitative studies could provide valuable insights into barriers, facilitators, and paediatric nurses’ lived experiences with resilience interventions.

## Strengths and limitations of the study

This systematic review systematically identified and appraised strategies to enhance resilience among paediatric nurses. Several limitations constrain the findings. The evidence base was small and heterogeneous, relying on only six studies with varying designs, outcomes, and intervention formats. Most studies relied on self-reported measures, which may be subject to bias. The geographical concentration of the studies in the USA also limits generalization to their healthcare systems. Additionally, inclusion criteria were restricted to English language, peer-reviewed publications, excluding grey literature sources such as theses and dissertations, which could introduce publication bias and lead to the omission of relevant evidence. Furthermore, study authors were not contacted to clarify missing or unclear data, which may have limited the comprehensiveness of the extracted information.

## Conclusion

Resilience-enhancing interventions such as mindfulness practices, education workshops, mHealth applications, and EAPs appear promising for improving the well-being of paediatric nurses. However, the current evidence base is limited and diverse, meaning these strategies should be considered provisional and applied with caution. Future multicentre longitudinal research is needed to establish their sustained effectiveness and develop interventions that are sustainable and easily integrated into paediatric healthcare practice.

## Supplementary Information

Below is the link to the electronic supplementary material.

Supplementary file1 (DOCX 14 KB)

Supplementary file2 (DOCX 16.8 KB)

Supplementary file3 (DOCX 15.8 KB)

## Data Availability

Not applicable.
