# Supplementary material for: Enhancing resilience and mental well-being among paediatric nurses: a systematic review of effective strategies and implementation challenges
Source: Eur J Pediatr. 2025 Nov 24;184(12):786. doi: 10.1007/s00431-025-06647-y (PMC12644147; doi:10.1007/s00431-025-06647-y)
Supplement: Supplementary file 1 — Supplementary file1 (DOCX 14 KB) [file 431_2025_6647_MOESM1_ESM.docx]

**Full electronical search string**

Scopus

("paediatric nurse" OR" pediatric nurse" OR “paediatric nurses” OR “pediatric nurses” OR "children's nurses" OR "paediatric nursing staff" OR “pediatric nursing staff”) AND ("resilience" OR “adaptability” OR “perseverance”) AND ("mental well-being" OR "mental wellness" OR “mental health”) AND ("strategies" OR "interventions" OR "programs" OR "support programs" OR "coping mechanisms")

Medline

("paediatric nurse" OR" pediatric nurse" OR “paediatric nurses” OR “pediatric nurses” OR "children's nurses" OR "paediatric nursing staff" OR “pediatric nursing staff”) AND ("resilience" OR “adaptability” OR “perseverance”) AND ("mental well-being" OR "mental wellness" OR “mental health”) AND ("strategies" OR "interventions" OR "programs" OR "support programs" OR "coping mechanisms")

PubMed

("paediatric nurse" OR" pediatric nurse" OR “paediatric nurses” OR “pediatric nurses” OR "children's nurses" OR "paediatric nursing staff" OR “pediatric nursing staff”) AND ("resilience" OR “adaptability” OR “perseverance”) AND ("mental well-being" OR "mental wellness" OR “mental health”) AND ("strategies" OR "interventions" OR "programs" OR "support programs" OR "coping mechanisms")

Web of Science

("paediatric nurse" OR" pediatric nurse" OR “paediatric nurses” OR “pediatric nurses” OR "children's nurses" OR "paediatric nursing staff" OR “pediatric nursing staff”) AND ("resilience" OR “adaptability” OR “perseverance”) AND ("mental well-being" OR "mental wellness" OR “mental health”) AND ("strategies" OR "interventions" OR "programs" OR "support programs" OR "coping mechanisms")

CINAHL

("paediatric nurse" OR" pediatric nurse" OR “paediatric nurses” OR “pediatric nurses” OR "children's nurses" OR "paediatric nursing staff" OR “pediatric nursing staff”) AND ("resilience" OR “adaptability” OR “perseverance”) AND ("mental well-being" OR "mental wellness" OR “mental health”) AND ("strategies" OR "interventions" OR "programs" OR "support programs" OR "coping mechanisms")

Embase

("paediatric nurse" OR" pediatric nurse" OR “paediatric nurses” OR “pediatric nurses” OR "children's nurses" OR "paediatric nursing staff" OR “pediatric nursing staff”) AND ("resilience" OR “adaptability” OR “perseverance”) AND ("mental well-being" OR "mental wellness" OR “mental health”) AND ("strategies" OR "interventions" OR "programs" OR "support programs" OR "coping mechanisms")

Google scholar

("paediatric nurse" OR" pediatric nurse" OR “paediatric nurses” OR “pediatric nurses” OR "children's nurses" OR "paediatric nursing staff" OR “pediatric nursing staff”) AND ("resilience" OR “adaptability” OR “perseverance”) AND ("mental well-being" OR "mental wellness" OR “mental health”) AND ("strategies" OR "interventions" OR "programs" OR "support programs" OR "coping mechanisms")
