# Supplementary material for: Enhancing resilience and mental well-being among paediatric nurses: a systematic review of effective strategies and implementation challenges
Source: Eur J Pediatr. 2025 Nov 24;184(12):786. doi: 10.1007/s00431-025-06647-y (PMC12644147; doi:10.1007/s00431-025-06647-y)
Supplement: Supplementary file 2 — Supplementary file2 (DOCX 16.8 KB) [file 431_2025_6647_MOESM2_ESM.docx]

**Table of excluded full-text studies**

| **Study (author, year)** | **Title** | **Reason for exclusion** |
| --- | --- | --- |
| White et al., 2023 | The Resilience of Pediatric Nurses in Context: A Mixed Methods Study | Full text not available |
| Lee et al., 2015 | Promoting Staff Resilience in the Pediatric Intensive Care Unit | Irrelevant to the research question |
| Robinson et al., 2022 | A Secondary Traumatic Stress Reduction Program in Emergency Room Nurses | Irrelevant to the research question |
| Liao et al., 2022 | Work stress, burnout, occupational commitment, and social support among Chinese pediatric nurses: A moderated mediation model | Full text not available |
| Martinez and Opalinski, 2019 | Building the Concept of Nurturing Resilience | Irrelevant to the research question |
| Goodman and Schorling, 2012 | A mindfulness course decreases burnout and improves well-being among healthcare providers | Irrelevant to the research question |
| Ramli et al., 2019 | Resilience among nurses working in paediatric wards in Brunei Darussalam: A qualitative study | Flawed methodology |
| Trygg et al., 2023 | Emergency Department Nurses' Experiences of a Mindfulness Training Intervention: A Phenomenological Exploration | Irrelevant to the research question |
| Anguis et al., 2023 | An Exploratory Study of ICU Pediatric Nurses' Feelings and Coping Strategies after Experiencing Children Death. Healthcare | Irrelevant to the research question |
| Lin et al., 2019 | The Effects of a Modified Mindfulness-Based Stress Reduction Program for Nurses: A Randomized Controlled Trial | Full text not available |
| Wong, 2021 | Exploring the Effect of Mindfulness on Burnout in a Pediatric Emergency Department | Flawed methodology |
| Zander et al 2013 | Exploring resilience in paediatric oncology nursing staff | Flawed methodology |
| Argyriadis et al., 2023 | Experimental Mindfulness Intervention in an Emergency Department for Stress Management and Development of Positive Working Environment | Irrelevant to the research question |
| Martinez and Opalinski, 2019 | Building the Concept of Nurturing Resilience | Irrelevant to the research question |
