# Supplementary material for: Enhancing resilience and mental well-being among paediatric nurses: a systematic review of effective strategies and implementation challenges
Source: Eur J Pediatr. 2025 Nov 24;184(12):786. doi: 10.1007/s00431-025-06647-y (PMC12644147; doi:10.1007/s00431-025-06647-y)
Supplement: Supplementary file 3 — Supplementary file3 (DOCX 15.8 KB) [file 431_2025_6647_MOESM3_ESM.docx]

**Detailed MMAT scoring table**

| **Study (Author/Year)** | **Study design** | **Q1. Clear research question** | **Q2. Data collection Methods appropriate** | **Q3. Sampling method appropriate** | **Q4. Measurement appropriate** | **Q5. Data analysis appropriate** | **Overall risk of bias** |
| --- | --- | --- | --- | --- | --- | --- | --- |
| Slater et al., 2018 | Clinical trial  (non-randomized) | yes | yes | Can’t tell | yes | yes | Moderate |
| Zadeh et al., 2012 | Clinical trial (non-randomized) | yes | yes | Can’t tell | yes | yes | moderate |
| Peterson et al., 2024 | 3-arm pre-post interventional | yes | yes | yes | yes | yes | low |
| Franco & Christie, 2021 | Quasi-experimental design | yes | yes | yes | yes | yes | low |
| Wei et al., 2020 | Cross-sectional (Qualitative descriptive study) | yes | yes | yes | yes | yes | low |
| Sadeghpour et al., 2021 | Clinical trial (RCT) | yes | yes | yes | yes | yes | low |
